# Magnesium oxide nanoparticles reduce clubroot by regulating plant defense response and rhizosphere microbial community of tumorous stem mustard (*Brassica juncea* var. *tumida*)

**DOI:** 10.3389/fmicb.2024.1370427

**Published:** 2024-03-20

**Authors:** Jingjing Liao, Zitong Yuan, Xiangmei Wang, Tingting Chen, Kun Qian, Yuanyuan Cui, Anping Rong, Chunyang Zheng, Yuanxiu Liu, Diandong Wang, Limei Pan

**Affiliations:** ^1^School of Advanced Agriculture and Bioengineering, Yangtze Normal University, Chongqing, China; ^2^College of Plant Protection, Southwest University, Chongqing, China

**Keywords:** MgO NPs, *Plasmodiophora brassicae*, soil microorganism, disease control, antioxidant enzyme

## Abstract

Clubroot, caused by *Plasmodiophora brassicae*, is a major disease that significantly impairs the yield of cruciferous crops and causes significant economic losses across the globe. The prevention of clubroot, especially in tumorous stem mustard (without resistant varieties), are is limited and primarily relies on fungicides. Engineered nanoparticles have opened up new avenues for the management of plant diseases, but there is no report on their application in the prevention of clubroot. The results showed that the control efficacy of 500 mg/L MgO NPs against clubroot was 54.92%. However, when the concentration was increased to 1,500 and 2,500 mg/L, there was no significant change in the control effect. Compared with CK, the average fresh and dry weight of the aerial part of plants treated with MgO NPs increased by 392.83 and 240.81%, respectively. Compared with the F1000 treatment, increases were observed in the content of soil available phosphorus (+16.72%), potassium (+9.82%), exchangeable magnesium (+24.20%), and water-soluble magnesium (+20.64%) in the 1,500 mg/L MgO NPs treatment. The enzyme-linked immune sorbent assay (ELISA) results showed that the application of MgO NPs significantly increased soil peroxidase (POD, +52.69%), alkaline protease (AP, +41.21%), alkaline phosphatase (ALP, +79.26%), urease (+52.69%), and sucrase (+56.88%) activities; And also increased plant L-phenylalanine ammonla-lyase (PAL, +70.49%), polyphenol oxidase (PPO, +36.77%), POD (+38.30%), guaiacol peroxidase (POX, +55.46%) activities and salicylic acid (SA, +59.86%) content. However, soil and plant catalase (CAT, −27.22 and − 19.89%, respectively), and plant super oxidase dismutase (SOD, −36.33%) activities were significantly decreased after the application of MgO NPs. The metagenomic sequencing analysis showed that the MgO NPs treatments significantly improved the α-diversity of the rhizosphere soil microbial community. The relative abundance of beneficial bacteria genera in the rhizosphere soil, including *Pseudomonas*, *Sphingopyxis*, *Acidovorax*, Var*iovorax*, and *Bosea*, was significantly increased. Soil metabolic functions, such as oxidative phosphorylation (ko00190), carbon fixation pathways in prokaryotes (ko00720), indole alkaloid biosynthesis (ko00901), and biosynthesis of various antibiotics (ko00998) were significantly enriched. These results suggested that MgO NPs might control clubroot by promoting the transformation and utilization of soil nutrients, stimulating plant defense responses, and enriching soil beneficial bacteria.

## 1 Introduction

Clubroot, caused by *Plasmodiophora brassicae*, is an economically important disease in Cruciferae crops worldwide (Dixon, 2009). In Fuling, China, clubroot is one of the main diseases in the production of tumorous stem mustard, which is the main factor threatening the development of the pickle industry. *P. brassicae* is an obligate parasite soil-borne plant pathogen, that causes root galls or clubs, resulting in water and nutrient absorption disorders, causing foliar wilting and even plant death, and ultimately, major yield losses in crop plants (Askarian et al., 2021). Management of clubroot is mainly through resistant varieties and chemical fungicides (Peng et al., 2014; Niemann et al., 2017). However, no resistant varieties have been cultivated so far for tumorous stem mustard, and only two active ingredients, fluazinam and cyazofamid, are registered in China. The strategies for clubroot control are very limited. Therefore, it is very important to explore different clubroot prevention techniques.

Nanoparticles (NPs), are particles in a nanorange structure (any dimension from 1 to 100 nm) with unique optical, magnetic, electrical, and thermal properties (Keck and Müller, 2013; Ojha et al., 2018). Due to their characteristics of improving drug delivery, slowing down the release of active ingredients, high efficiency in lower doses, and ecological friendliness, have received increasing attention for their potential applications in plant disease control (Malandrakis et al., 2019). At present, a range of inorganic and organic nanomaterials have been developed and demonstrated to have significant antibacterial, antifungal, and antiviral properties *in vitro*, in the greenhouse, and the field, such as TiO_2_, CuO, Zn, ZnO, Fe, Mg, MgO, Mg(OH)_2_, Al, Si, Ag, carbon and other nanomaterials (Chen et al., 2020). Liang et al. (2022) showed that the incidence index of tomato bacterial wilt after treated with berberine-loaded ZnO-Z nanomaterial was 45.8%, significantly lower than in the control treatment (94.4%). Ahmed et al. (2022) showed that spraying 250 mg/L of chitosan-Fe composite nanomaterials on rice leaves could reduce the incidence of rice white leaf blight by 67.1%. However, there is no report on the prevention of clubroot by nanomaterials.

MgO NPs, as a metal oxide nanomaterial, have been recognized as safe material by the US Food and Drug Administration (21CFR184.1431) (Cai et al., 2018; Ahmed et al., 2021), possessing unique physical and chemical properties of nanomaterials (small size, crystal structure, stability, specific surface area, etc.) (Ahmed et al., 2021). MgO NPs have a wide range of applications in various fields, such as water pollution treatment (Uko et al., 2022), drug delivery (Nejati et al., 2022), food packaging bags (Swaroop and Shukla, 2018), and antibacterial applications (Cai et al., 2018; Chen et al., 2020). They are non-toxic, biocompatible, and environmentally friendly materials. Studies have shown that MgO NPs could inhibit a variety of plant pathogens, such as *Ralstonia solanacearum*, *Fusarium oxysporum*, *Phytophthora nicotianae*, and *Thielaviopsis basicola* (Cai et al., 2018; Abdel-Aziz et al., 2020; Chen et al., 2020; Ahmed et al., 2021). Currently, it is unknown whether MgO NPs can be used for the prevention of clubroot. However, there have been no reports on the effect of MgO NPs on clubroot so far.

The mechanism of plant disease control by nanomaterials could include several aspects. Firstly, nanomaterials can directly act on pathogens, causing cell damage, stimulating reactive oxygen species (ROS) accumulation, and destroying cell proliferation. Chen et al. (2020) proved that MgO NPs damaged the bacterial structure, caused oxidative stress and inhibited the growth and reproduction of *P. nicotianae* and *T. basicola* through direct contact with pathogens. Cai et al. (2018) showed that MgO NPs directly attached to the surface of bacteria, caused physical damage to the cell membrane, reduced its motility and biofilm formation ability, stimulated ROS accumulation, and induced DNA damage. Secondly, nanomaterials can enhance plant disease resistance by improving plant growth, photosynthetic capacity, and plant hormones. Ahmed et al. (2022) demonstrated that BNCs (bioengineered chitosan-iron nanocomposites) significantly reduced the BLB (bacterial leaf blight) disease incidence through modulation of antioxidant enzymes, promotion of photosynthesis efficiency and nutritional profile of rice plants. Imada et al. (2016) demonstrated that MgO NPs induced resistance of susceptible varieties’ roots and hypocotyl to bacterial wilt by activating salicylic acid (SA), jasmonic acid (JA), and ethylene (ET) signaling pathways in tomatoes. Thirdly, nanomaterials can alter the plant rhizosphere microbial community and create a microbial environment that is not conducive to the development of diseases (Yan et al., 2020; Liu et al., 2023). Rhizosphere microorganisms play an important role in promoting plant growth, stress tolerance, and pathogen resistance (Williams et al., 2023). The invasion of bacterial and fungal pathogens caused significant changes in plant rhizosphere microbial community structure, which may be the favorable microbial environment conditions created by pathogenic microorganisms for successful infection (Xi et al., 2023). The application of nanomaterials can destroy the favorable microbial environmental conditions for pathogen infection by regulating the structure of plant rhizosphere microbial community, to achieve the purpose of disease control. Ahmed et al. (2022) revealed that BNCs amendment reshaped the phyllospheric and root-endophytic bacterial community of rice, and increased the bacterial community diversity in healthy and diseased plants, which resulted in the decreasing of *Xanthomonas* pathogen.

Therefore, our objective in this study was to investigate (1) the effect of MgO NPs on tumorous stem mustard resistance to clubroot; (2) the physiological and biochemical, and soil microbe bases of MgO NPs improvement of this resistance to clubroot. This study will provide insights into the mechanism of MgO NPs on crop resistance to clubroot from the perspectives of soil nutrition, plant defense levels, and rhizosphere microbial health. It aims to fill the research gaps in the prevention of clubroot using nanomaterials, and provide new research ideas for the control of this global soil-borne disease.

## 2 Materials and methods

### 2.1 Production and characterization of MgO NPs

Magnesium nitrate hexahydrate (Mg(NO_3_)_2_·6H_2_O, 0.028 mol) was dissolved in 70 mL anhydrous ethanol, followed by the successive addition of 5 mL ethylenediaminetetraacetic acid disodium salt (0.023 mol·L^−1^) and 5 mL hydrazine hydrate (80%) with thoroughly stirring. Then, the solution was transferred into a polyphenylene-lined kettle of 100 mL capacity and incubated at 200°C for 20 h. After the reaction, the white suspension in the reaction kettle was poured out and washed several times until the pH of the supernatant reached a neutral level. The white precipitate was dried at 60°C for 6 h, and the sample was calcined at 500°C for 5 h in an air atmosphere.

The morphology of MgO NPs was assessed by scanning electron microscope (SEM; Hitachi SU8100). The basic and surface structures information of MgO NPs were conducted by K-Alpha+X-ray photoelectron spectroscopy (XPS, Thermo Fisher) and Fourier transformed infrared spectra (FTIR, IRAffinity-1). The powder X-ray diffraction (XRD) patterns (Cu Kα) were recorded by Rigaku Smartlab SE with scanned in step of 1° (2*θ*) in the range from 20° to 80°. Zeta potential of MgO NPs was measured using Malvern ZEV3600.

### 2.2 Experimental design

To evaluate the greenhouse control effect of different concentrations of MgO NPs on the clubroot of tumorous stem mustard (the cultivar “Fuza No. 2,” susceptible to *P. brassicae*), we set three concentration gradients of MgO NPs (500, 1,500, 2,500 mg/L), with 1,000-fold dilution of fluazinam (F1000) and water treatment (CK) after inoculation of *P. brassicae* as controls. Each treatment was performed with three repetitions, and each repetition contained about 20 plants. Seed treatment and preparation of resting spore suspension were refer to Liao et al. (2022). The greenhouse with a constant temperature of 24°C and natural humidity, under a 16/8 h alternating light and dark cycle. Turf soil and vermiculite were mixed at a volume ratio of 1:3 and sterilized at 121°C for 20 min, which was used as a growth substrate of tumorous stem mustard. Three weeks after sowing, each plant was inoculated with 2 mL resting spores (1 × 10^8^ mL^−1^). At 1 d, and 11 d after inoculation, each plant was treated with 10 mL MgO NPs (500, 1,500, 2,500 mg/L), F1000, and water, respectively. The severity of clubroot was investigated at 4 weeks after inoculation, as described by Agarwal et al. (2011), using a scale of 0 to 4, where 0 = no symptoms, 1 = small and few galls, mainly on the fibrous root, 2 = small and few galls, mainly on the taproot, 3 = medium galls on the taproot and fibrous roots, 4 = large galls or decayed. Disease index (DI) was calculated using the following formula: DI = ∑ (n0 × 0 + n1 × 1 + n2 × 2 + n3 × 3 + n4 × 4) × 100 / (N × 4). Where n0 to n4 are the number of plants corresponding to symptom severity scores 0 to 4, and N is the total number of plants. In addition, the agronomic characteristics such as stem diameter, number of leaves, maximum leaf area, fresh weight of aerial part, fresh weight of root, and dry weight of aerial part were investigated. The stem thickness was measured at 0.5 cm above the cotyledon node using an electronic Vernier caliper. The maximum leaf area was measured by ImageJ software after taking photos. During the investigation of the number of plant leaves, leaves measuring less than 1 cm in length were excluded from consideration.

To evaluate the effects of MgO NPs on soil physicochemical property and soil bacterial community, field soil (collected from 0 to 20 cm surface soil of an agricultural field in Fuling, Chongqing, 29°57′28″ N and 107°31′31″ E) was used to grow tumorous stem mustard. We set five treatments, including three concentration gradients of MgO NPs treatments, F1000 treatment, and CK treatment. Each treatment was performed with six repetitions, and each repetition contained 12 plants. After 4 weeks of inoculation, the seedlings were pulled out, and the soil on the root was shaken off as bulk soil. Then the roots were washed with 30 mL sterile phosphate buffer (PBS, pH 7.0, 0.5 mM) for 3–5 min. After repeated cleaning for three times, a total of 90 mL PBS solution was collected. After centrifugation at 8000 rpm for 5 min, the precipitation was the rhizosphere soil. After freeze-drying (Castaño et al., 2016; Weißbecker et al., 2017), part of the rhizosphere soil was sent to BGI company for metagenomic sequencing, and the other part was used for soil enzyme activity determination. The roots of the cleaned plants were stored at −80°C to determine the activity of the antioxidant enzyme and the content of SA.

### 2.3 Detection of enzyme activity and SA content in soil and plant samples

Enzyme activity and SA content were detected with ELISA (enzyme-linked immune sorbent assay) kits by Jiangsu Meimian Industrial Co., Ltd. (Xu et al., 2019). The PBS solution (9 mL, pH 7.4) was added into a soil sample (1 g). After homogenization, the mixture was centrifuged at 4°C for 20 min at 3000 rpm. The supernatant was the sample to be tested. A fresh plant tissue sample (0.1 g) was fully grinded in liquid nitrogen, and 1 mL extraction solution (80% methanol) was added. After being treated overnight at −20°C, the mixture was centrifuged at 4°C for 1 h at 8000 rpm. After passing through a C-18 solid phase extraction column, the supernatant was dried under vacuum or with nitrogen gas. Before the determination, 1 mL of PBS (pH 7.4) was added to the dry powder and mixed well. After being treated at room temperature for 30 min, the mixture was centrifuged at 4°C for 15 min at 8,000 rpm, and the supernatant was the sample to be tested.

The soil or plant supernatants (50 μL) and the standard products (50 μL) were added into the wells that had been previously coated with catalase (CAT), peroxidase (POD), L-phenylalanine ammonia-lyase (PAL), super oxidase dismutase (SOD), polyphenol oxidase (PPO), peroxidase (POX), alkaline protease (AP), alkaline phosphatase (ALP), urease, sucrase, and SA antibodies, respectively. And then the horseradish peroxidase (HRP)-conjugate reagent (100 μL) was added, except the blank wells. The 96-well plate was covered with an adhesive strip and incubated at 37°C for 60 min. Each well was aspirated and washed with wash solution (400 μL) for five times. After the last wash, any remaining wash solution was removed. The plate was then inverted and blotted against clean paper towels. Chromogen solution A (50 μL) and B (50 μL) were added to each well and gently mixed. The plate was then incubated at 37°C for 15 min. Plates were protected from light. Finally, stop solution (50 μL) was added to each well. The color of the wells should change from blue to yellow. The deeper the color, the higher the enzyme activity. The OD value was measured at 450 nm using a microtiter plate reader within 15 min. Six repetitions were set in each treatment. The standard curve was used to determine the enzyme activity of all samples. Each standard curve contained six concentrations, with two repetitions. For details, refer to the kit instructions.

### 2.4 Measurement of soil physicochemical properties

The physicochemical properties of bulk soil samples were measured by Convinced-Test Company (Nanjing, China). Electrical conductivity (EC) (electrode method, HJ 802–2016), pH (glass electrode method, NY/T 1121.2–2006), alkaline nitrogen (alkali-diffusion method, LY/T 1229–1999), available phosphorus (molybdenum-antimony resistance colorimetric method, NY/T 1121.7–2014), available potassium (flame spectrophotometry), total magnesium (Mg) (inductively coupled plasma mass spectrometry, DZT 0279.2–2016), exchangeable Mg (ammonium acetate method, NY/T 1121.13–2006), and water-soluble Mg (ICP-OES) were detected.

### 2.5 DNA extraction, sequencing, and microbial community analysis

Genomic DNA was extracted from the soil samples using the commercial DNA extraction kit (DP336, TIANGEN BIOTECH CO., LTD, Beijing). The DNA concentration was measured by a microplate reader, and the DNA integrity was detected by agarose gel electrophoresis. After passing the quality inspection, the DNA was interrupted by a Covaris ultrasonic crusher, 200–400 bp bands were enriched by magnetic beads, then PCR amplification was performed and the amplified products were recovered. The PCR product is denatured into a single strand and cyclized, and after the uncyclized DNA molecule is digested, the qualified library can be sequenced on the machine. The DNBSEQ T7 platform was used for sequencing. SOAPnuke software (Chen et al., 2018) was used for filtering the original data. Bowtie 2 (Langmead and Salzberg, 2012) was used to compare the host sequence and remove the host sequence to generate clean data. MEGAHIT (Li et al., 2015) was used to assemble the sequences based on k-mer to generate contigs. Then MetaGeneMark (Zhu et al., 2010) was used to predict the gene sequences in contigs. CD-HIT software (Fu et al., 2012) was used to remove the redundancy of the obtained genes, and Salmon software (Patro et al., 2017) was used to count the relative abundance of each gene. DIAMOND (Buchfink et al., 2015) was used to compare non-redundant genes to eggNOG, KEGG, BacMet, CARD, COG, CAZy, and Swiss-prot databases to complete gene function annotation. The sequence number of species contained in the samples was calculated by Kraken2 and blasting with the NCBI NT database, and then the actual abundance of species was estimated by Bracken2 to complete the species annotation.

Chao 1, Shannon index, and Simpson were calculated to evaluate the α-diversity. Principal coordinate analysis (PCoA) and non-metric multidimensional scaling (NMDS) based on bray-curtis distance dissimilarities were constructed to evaluate β-diversity. The enrichment heatmap of differential ko (KEGG orthology) was performed using the Omic-Share online platform.^1^

### 2.6 Statistical analysis

General statistical analysis was performed by IBM SPSS Statistics 22. Data of disease severity, plant growth, soil property, and enzyme activity were analyzed by one-way analysis of variance (one-way ANOVA, LSD, *p* < 0.05).

## 3 Results

### 3.1 Characterization of MgO NPs

**Figure 1:**
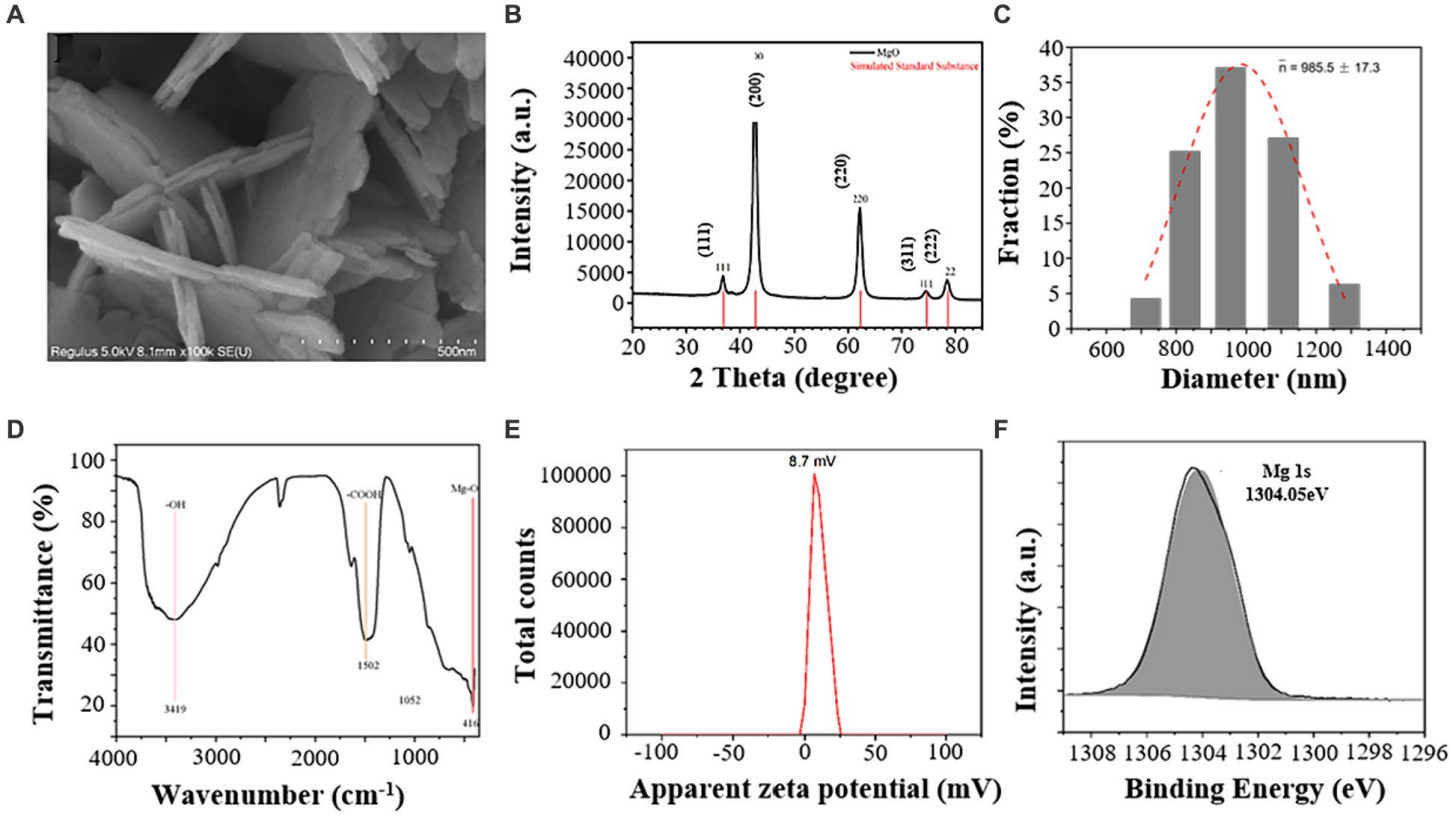
Characterization of magnesium oxide nanoparticles (MgO NPs). **(A)** SEM (Scanning Electron Microscope) analysis of MgO NPs. **(B)** XRD (X-Ray Diffraction) pattern of MgO NPs. **(C)** Hydraulic diameters of MgO NPs. **(D)** FTIR (Fourier Transform Infrared Spectrometer) spectra of MgO NPs. **(E)** Zeta potential of MgO NPs. **(F**) XPS (X-ray photoelectron spectroscopy) of the Mg 1 s spectrum in MgO NPs.

MgO NPs were prepared by hydrothermal synthesis method. Scanning electron microscopy (SEM) results showed that MgO NPs were flaky with an average thickness of 21.8 nm (Figure 1A). X-ray diffraction (XRD) results showed that MgO NPs were in complete agreement with the characteristic peaks of its reference materials (Figure 1B). The hydraulic diameters of MgO NPs were 985.5 ± 17.3 nm (Figure 1C). The surface functional groups of MgO NPs measured by Fourier infrared spectroscopy (FTIR) showed that MgO NPs had characteristic peaks at 3419, 1502, and 416 cm^−1^, corresponding to –OH, -COOH, and Mg-O, respectively (Figure 1D). The zeta potential of MgO NPs in water was 8.7 mV (Figure 1E), indicating that the low stability of the NPs in water. It mainly due to the partial hydrolysis of the carboxyl group, resulting in a decrease in zeta potential. Figure 1F focused on Mg 1 s region of MgO NPs, and which showed a peak at 1304.05 eV, characteristic of Mg^2+^ species in MgO.

### 3.2 Effect of MgO NPs on clubroot control, antioxidant enzyme activity, and SA accumulation

**Figure 2:**
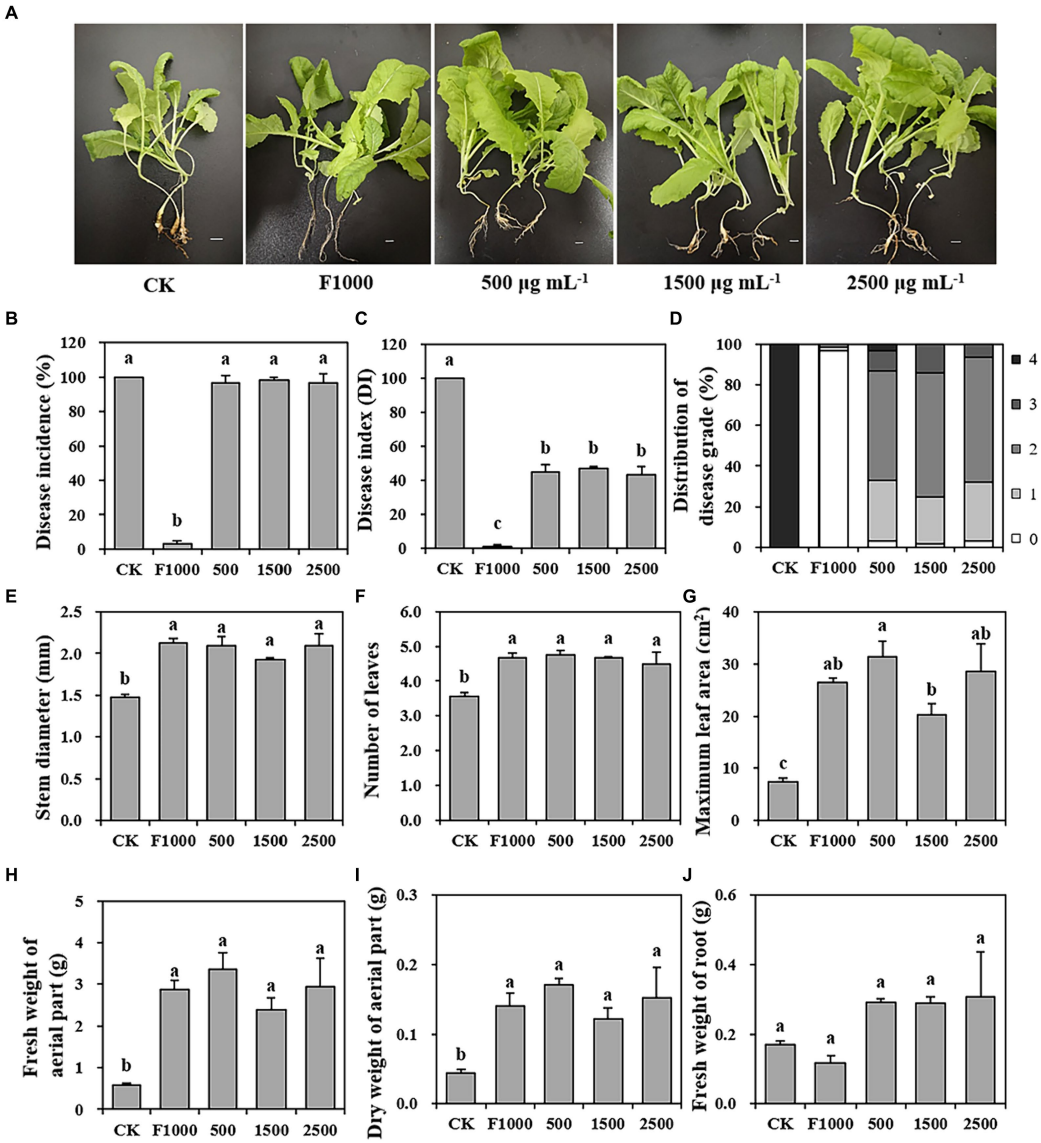
Effect of MgO NPs on the clubroot incidence, disease index, and plant growth at three different concentrations. **(A)** Phenotype of plants with different treatments. **(B–D)** Clubroot severity of tumorous stem mustard under different treatments. **(E–J)** Plant growth under different treatments. All data are represented with a mean of three replicates ± standard error. Different lowercase letters represent the significant differences at *p* < 0.05 level (one-way ANOVA, LSD). The ruler in **(A)** represents 1 cm. 0–4 in **(D)** represent five classes of scale which were used to calculate the disease index (DI) according to Agarwal et al. (2011). CK, F1000, 500, 1,500, and 2,500 represent water treatment, 1,000-fold dilution of fluazinam treatment, 500, 1,500, and 2,500 mg/L concentration of MgO NPs treatment after the inoculation of *P. brassicae*, respectively.

To evaluate the disease-suppressing potential of MgO NPs against clubroot, we investigated and calculated the disease incidence, disease index (DI), and distribution of disease grade of clubroot on tumorous stem mustard at 4 weeks after inoculation. The results showed that the severity of clubroot was significantly decreased after being treated with MgO NPs (Figures 2A–D). The disease incidences in MgO NPs treatments (500, 1,500, and 2,500 mg/L) showed no significant differences compared to CK (100%). However, it is important to note that DI in MgO NPs treatments were 45.08, 46.84, and 43.18, respectively, which were significantly lower than CK (100). The average control efficiency reached 54.97%. Compared to CK (100% grade 4 galls), MgO NPs treatments led to a significant reduction in grade 4 galls, with the lowest proportion at 3.28, 0.00, and 0.00%. F1000 treatment exhibited the most effective control of clubroot, with a disease incidence and DI of 3.33% and 1.25, respectively. There was no significant difference among the three MgO NPs concentrations in disease incidence and DI, which were significantly higher and lower than F1000 and CK treatment, respectively. Furthermore, the application of MgO NPs had a notable positive impact on plant growth compared with CK, as indicated by significant increases in stem diameter (38.18%), number of leaves (29.87%), maximum leaf area (264.65%), fresh weight of aerial part (392.83%), and dry weight of aerial part (240.81%) (Figures 2E–I). There was no significant difference in plant growth between F1000 and MgO NPs treatments.

**Figure 3:**
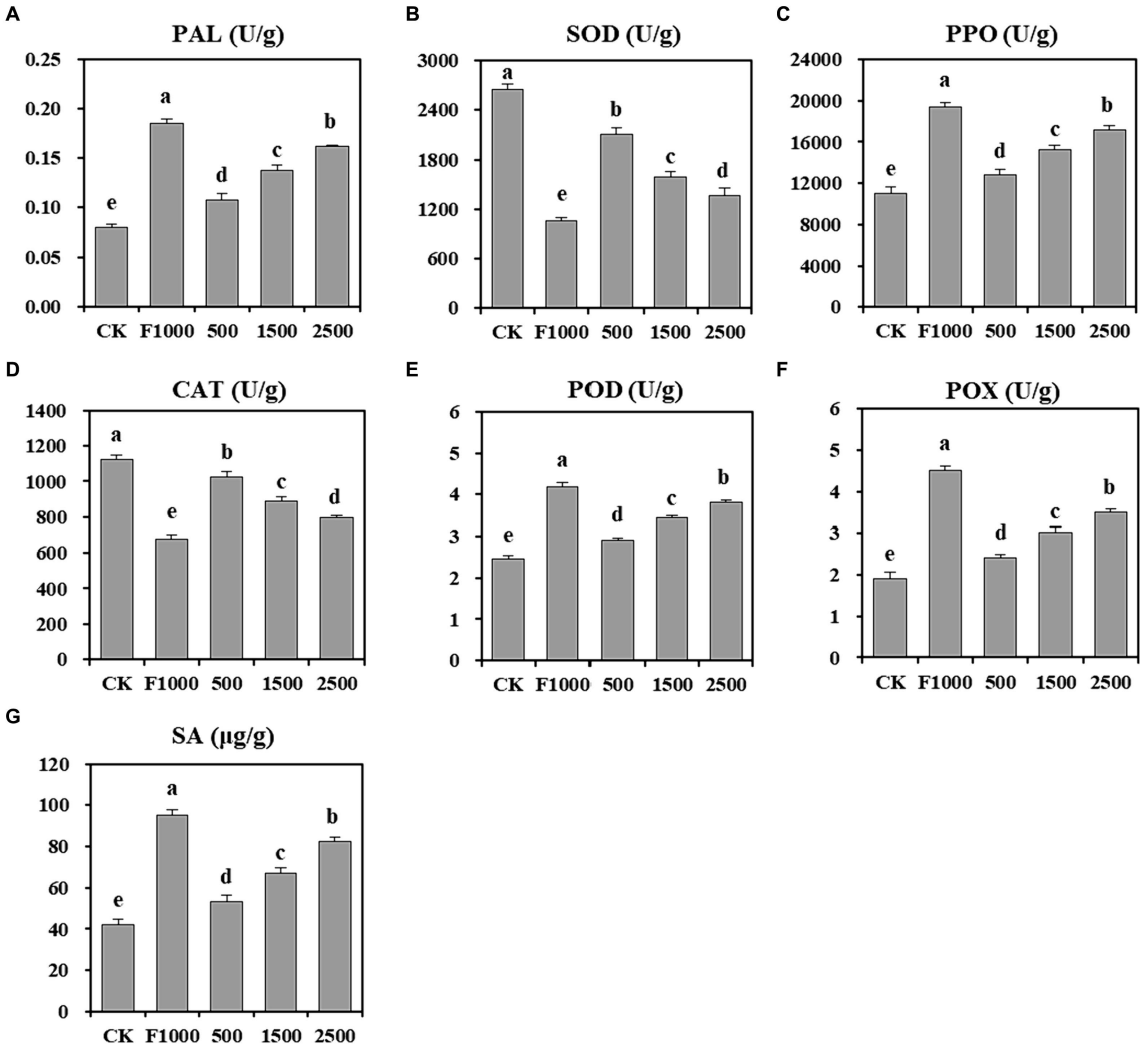
Effect of MgO NPs on the plant defense enzyme activity and the content of SA. **(A)** L-Phenylalanine ammonla-lyase (PAL). **(B)** Super oxidase dismutase (SOD). **(C)** Polyphenol oxidase (PPO). **(D)** Catalase (CAT). **(E)** Peroxidase (POD). **(F)** Guaiacol peroxidase (POX). **(G)** Salicylic acid (SA). U/g represents enzyme activity unit per gram of root samples. All data are represented with a mean of six replicates ± standard error. Different lowercase letters represent the significant differences at *p* < 0.05 level (one-way ANOVA, LSD). CK, F1000, 500, 1,500, and 2,500 represent water treatment, 1,000-fold dilution of fluazinam treatment, 500, 1,500, and 2,500 mg/L concentration of MgO NPs treatment after the inoculation of *P. brassicae*, respectively.

The application of MgO NPs also significantly stimulated the activity of antioxidant enzymes, and promoted the accumulation of SA in plants (Figure 3). The activities of PAL, PPO, POD, and POX were significantly increased in the MgO NPs treatments compared to the CK, and these enzyme activities demonstrated a further enhancement with increasing MgO NPs concentration. Compared with CK, the average enzyme activities increased by 70.49, 36.77, 38.30, and 55.46%, respectively. However, they were significantly lower than those observed in the F1000 treatment. SOD and CAT activities in MgO NPs treatments were significantly decreased compare with CK. As the concentration of MgO NPs increased, the enzyme activities decreased. When the concentration of MgO NPs reached 2,500 mg/L, the SOD and CAT activities were the lowest, representing a 41.86 and 29.29% decrease compared with CK, respectively. However, these two enzyme activities were significantly higher than those observed in the F1000 treatment. Moreover, the content of SA in plants treated with MgO NPs at concentration of 500, 1,500, and 2,500 mg/L were 53.35, 66.92, and 82.53 μg/g, respectively, which were significantly higher than that observed in the CK (42.29 μg/g). Additionally, the SA content increased with the increasing concentration of MgO NPs. However, it was significantly lower than that observed in the F1000 treatment (95.22 μg/g). These results indicate that MgO NPs could increase the activity of defense enzymes and the content of SA in plants.

### 3.3 Effect of MgO NPs on soil physicochemical properties

**Figure 4:**
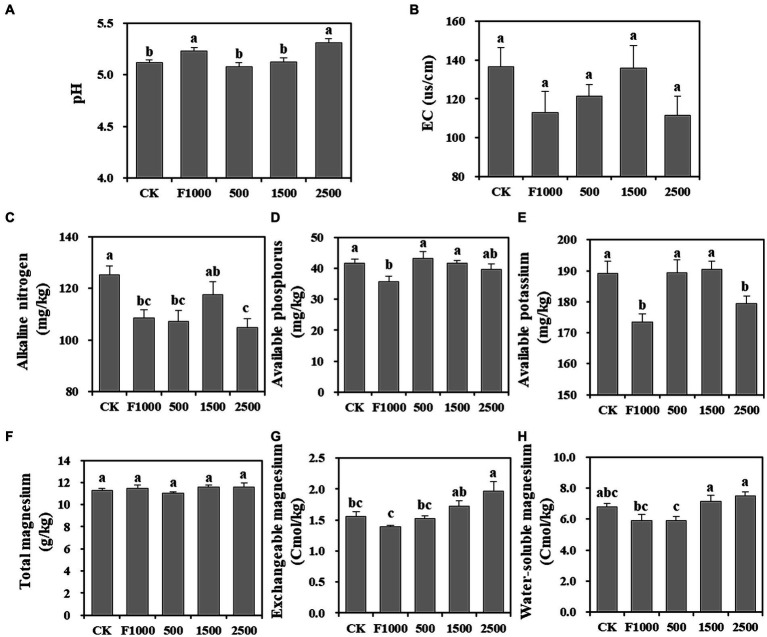
Effect of MgO NPs on the physicochemical properties of soil samples. **(A)** Soil pH. **(B)** Electric conductivity (EC). **(C–E)** Soil fertility index, alkaline nitrogen, available phosphorus, and available potassium. (**F–H**) The content of different forms of magnesium in soil. All data are represented with a mean of six replicates ± standard error. Different lowercase letters represent the significant differences at *p* < 0.05 level (one-way ANOVA, LSD). CK, F1000, 500, 1,500, and 2,500 represent water treatment, 1,000-fold dilution of fluazinam treatment, 500, 1,500, and 2,500 mg/L concentration of MgO NPs treatment after the inoculation of *P. brassicae*, respectively.

After 4 weeks of inoculation, the pH of bulk soil in F1000 and 2,500 mg/L MgO NPs treatment was significantly higher than that of other treatments, but the pH in all treatments varied from 5.08 to 5.31, which was suitable for the infection of *P. brassicae* (Figure 4A). There was no significant difference in EC in all treatments (Figure 4B).

The contents of alkaline nitrogen, available phosphorus, and available potassium in soil can reflect soil fertility (Figures 4C–E). The content of these three elements was highest in CK, which may be due to the formation of clubroot affecting the absorption of soil nutrients by roots. In contrast, these three elements had the lowest content in the F1000 treatment which showed the lowest severity of clubroot. The average contents of these three elements in MgO NPs treatments increased by 1.25, 16.08, 7.48%, respectively, compared with the F1000 treatment. The difference was less or not significant (1,500 mg/L MgO NPs) compared with CK.

The results of Mg content showed that there was no significant difference in total Mg content in all treatments. The exchangeable Mg content significantly increased by 26.46 and 41.55% when comparing the 2,500 mg/L MgO NPs treatment with CK and F1000, respectively. The water-soluble Mg content significantly increased by 20.64 and 26.69% in the 1,500 and 2,500 mg/L MgO NPs treatments, respectively, as compared to F1000 (Figures 4F–H). Soil exchangeable and water-soluble Mg are forms of available Mg, and their content directly reflects the status of soil Mg supply. Therefore, the application of MgO NPs could improve soil Mg supply.

### 3.4 Soil enzyme activity was stimulated by MgO NPs

**Figure 5:**
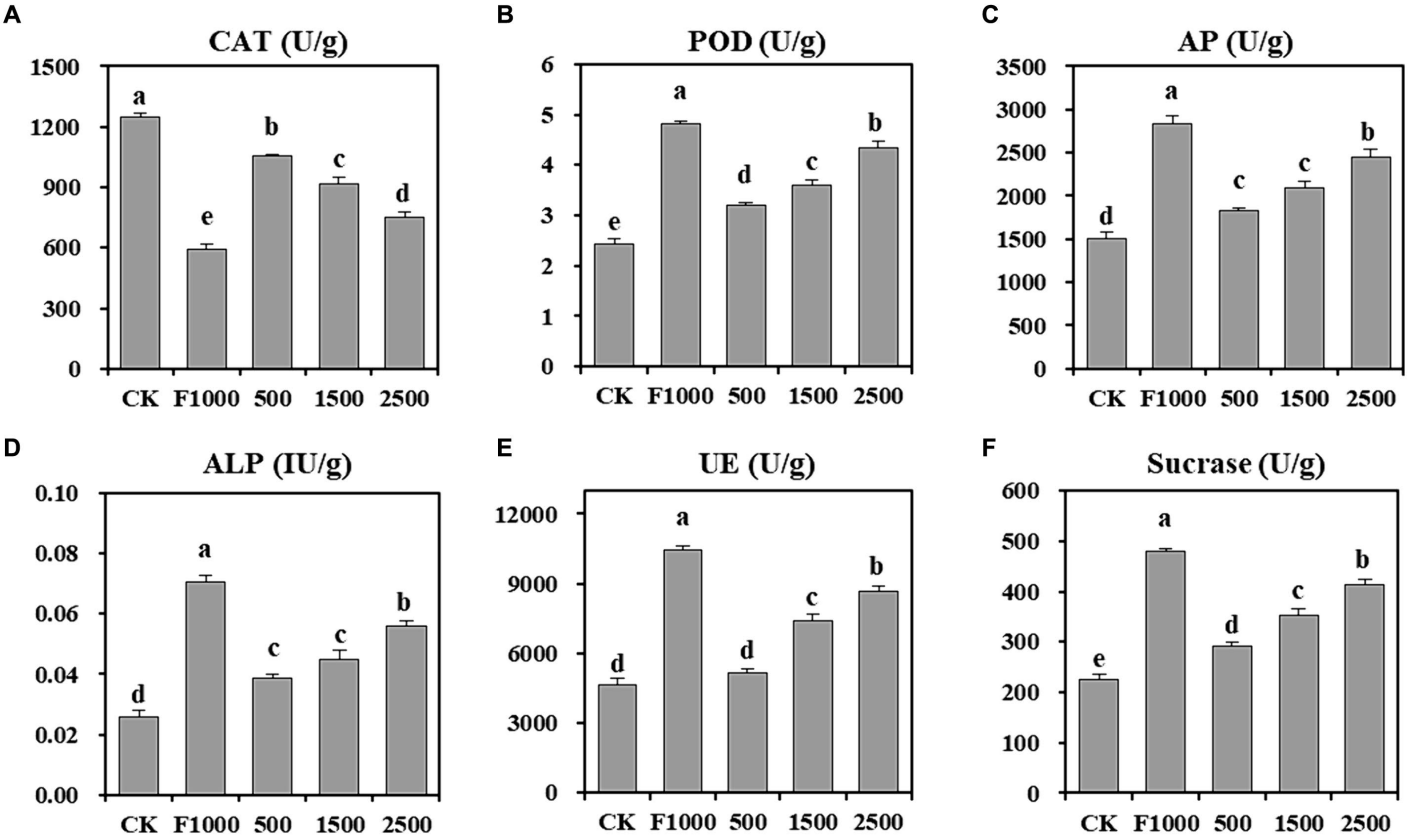
Effect of MgO NPs on the soil enzyme activity. **(A)** Catalase (CAT). **(B)** Peroxidase (POD). **(C)** Alkaline protease (AP). **(D)** Alkaline phosphatase (ALP). **(E)** Urease. **(F)** Sucrase. U/g represent enzyme activity unit per gram of soil samples. All data are represented with a mean of six replicates ± standard error. Different lowercase letters represent the significant differences at *p* < 0.05 level (one-way ANOVA, LSD). CK, F1000, 500, 1,500, and 2,500 represent water treatment, 1,000-fold dilution of fluazinam treatment, 500, 1,500, and 2,500 mg/L concentration of MgO NPs treatment after the inoculation of *P. brassicae*, respectively.

Soil enzymes play a crucial role in the soil bioprocesses, like degradation and mineralization of organic compounds, circulation of nutrients. Their response to environmental disturbances makes them a potential indicator of soil microbiological quality. Hence, assessing soil enzyme activity aids in evaluating the impact of nanoparticles on soil nutrient metabolism and soil microbial processes (Asadishad et al., 2018). The results showed that (Figure 5) the activity of POD, AP, ALP, urease, and sucrase were significantly increased at 500 (31.98, 21.51, 49.85, 11.27, and 29.20%, respectively), 1,500 (47.71, 39.28, 72.81, 59.36, and 57.05%, respectively), and 2,500 mg/L (78.34, 62.82, 115.11, 87.43, and 84.38%, respectively) MgO NPs treatments compared with CK. However, they were significantly lower than that observed in the F1000 treatment. The activity of CAT showed the opposite trend, which was significantly decreased at 500 (15.51%), 1,500 (26.22%), and 2,500 mg/L (39.95%) MgO NPs treatments and the F1000 treatment (52.72%) compared with CK.

### 3.5 Soil microbial α-diversity increased and β-diversity was significantly altered after the application of MgO NPs

Sequencing of the total DNA of 15 soil samples revealed 2,077,196 contigs, 2,814,529,366 bp assembly length, and 3,183,180 de-duplicated CDS, and the average values were 138,480, 187,635,291, and 227,331, respectively. After species annotation and abundance analysis of clean data, microbial species composition, diversity, significant differences were visualized. After functional annotation and abundance analysis of non-redundant genes, the data were also visualized.

**Figure 6:**
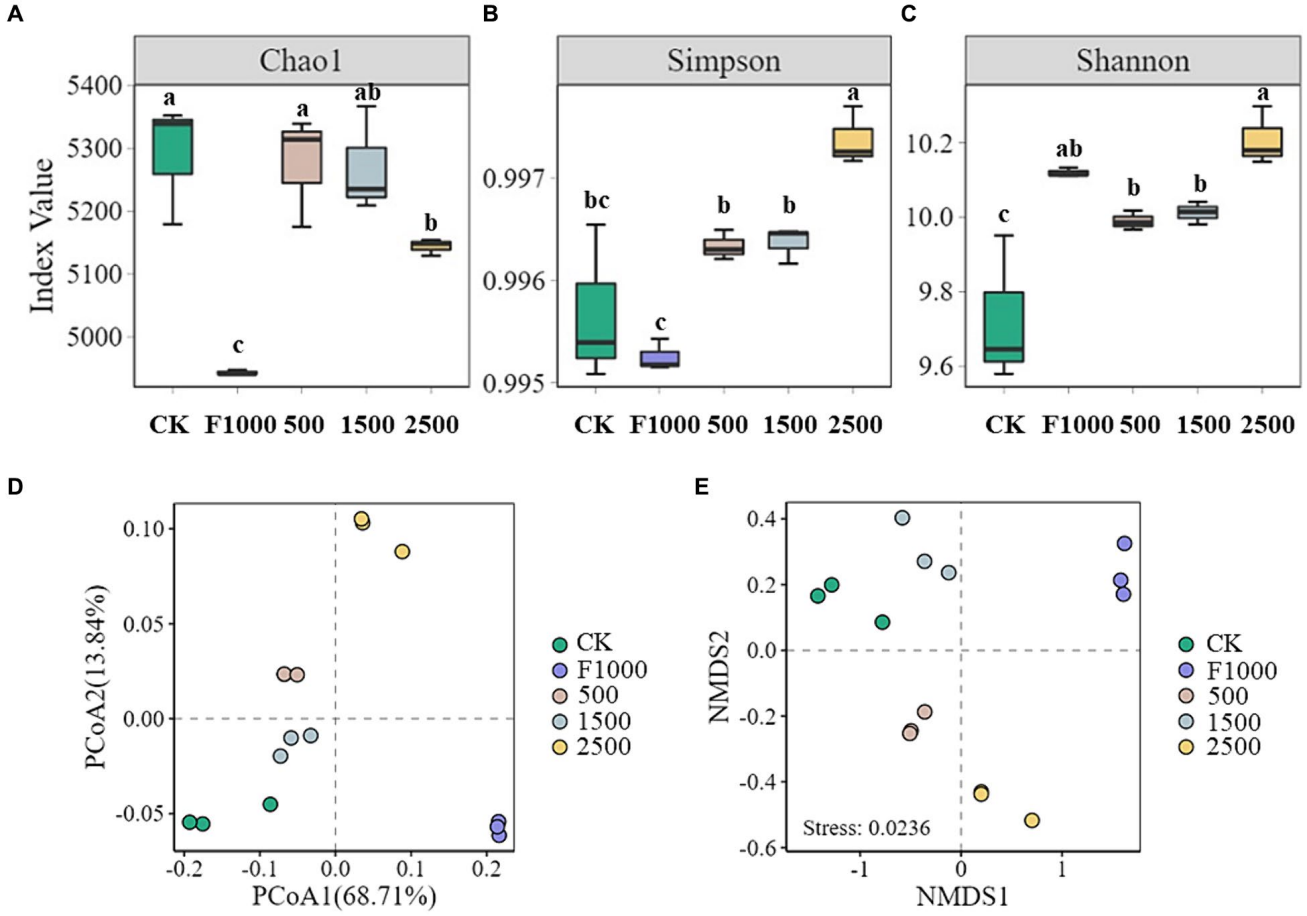
Effect of MgO NPs on α- and β-diversity of soil microbial community. **(A–C)** α-diversity indices, Chao1, Simpson, and Shannon. All data are represented with a mean of three replicates ± standard error. Different lowercase letters represent the significant differences at *p* < 0.05 level (one-way ANOVA, LSD). **(D)** Principal coordinate analysis (PCoA) of the microbial community. **(E)** Non-metric multidimensional scaling analysis (NMDS) of the microbial community. The analysis was conducted based on the Bray-Curtis distance. CK, F1000, 500, 1,500, and 2,500 represent water treatment, 1,000-fold dilution of fluazinam treatment, 500, 1,500, and 2,500 mg/L concentration of MgO NPs treatment after the inoculation of *P. brassicae*, respectively.

The α-diversity analysis evaluates microbial species richness and diversity within each soil sample on three indices: Chao 1, Shannon, and Simpson. The application of MgO NPs significantly increased the α-diversity in rhizosphere soil of diseased tumorous stem mustard as compared with F1000 and CK treatments (Figures 6A–C). The Chao 1 and Simpson indices in MgO NPs treatments were significantly higher than those of in F1000 treatment, and the Simpson and Shannon indices were significantly higher in 2,500 mg/L MgO NPs treatment than those of in CK treatment. There was no significant difference in α-diversity between 500 and 1,500 mg/L MgO NPs treatments, but Simpson and Shannon indices were significantly lower than those of in 2500 mg/L MgO NPs treatment.

Significant differences in microbial community composition in different treatments revealed by PCoA and NMDS (Figures 6A–C). The first principal coordinate (PCoA1) and the second principal coordinate (PCoA2) showed 68.71 and 13.84% of the total variation in the microbial community, respectively, based on bray-crutis dissimilarity. The results of NMDS (non-metric multidimensional scaling) analysis showed that the stress value was 0.0236 (< 0.05), which could accurately reflect the significant difference in microbial community composition among different treatments (Figures 6D,E).

### 3.6 Mgo NPs reshaped the microbial community in the rhizosphere soil of tumorous stem mustard

**Figure 7:**
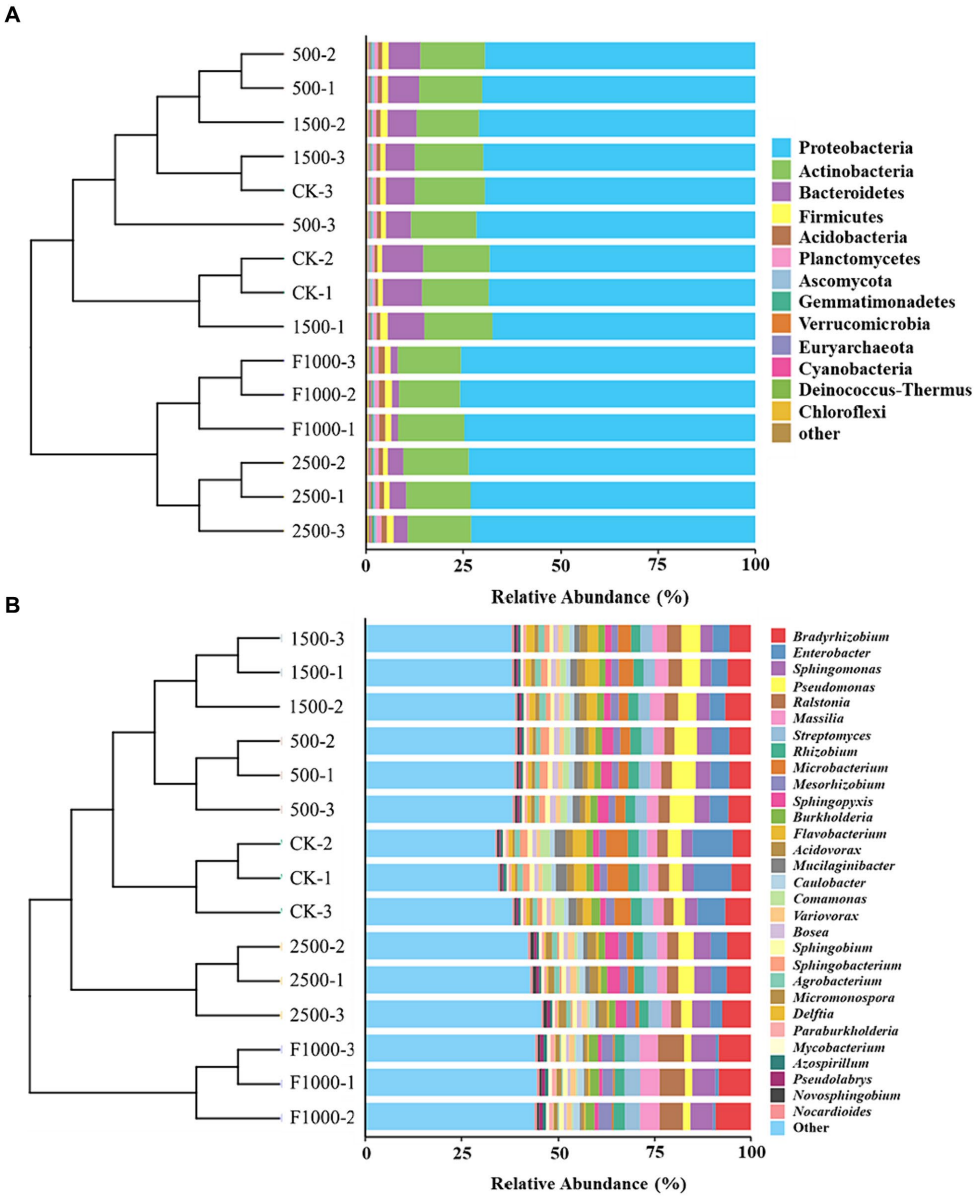
Relative abundance of dominant phyla and genera in different treatments. **(A)** Phylum level. **(B)** Genus level. The analysis was conducted based on the Bray-Curtis distance. CK, F1000, 500, 1,500, and 2,500 represent water treatment, 1,000-fold dilution of fluazinam treatment, 500, 1,500, and 2,500 mg/L concentration of MgO NPs treatment after the inoculation of *P. brassicae*, respectively. -1, -2, and -3 represent three replicates in each treatment.

The predominant phyla with an average relative abundance (> 1%) were Proteobacteria (68.67–75.35%), Actinobacteria (16.35–17.43%), Bacteroidetes (1.81–9.40%), Firmicutes (1.27–1.67%), Acidobacteria (0.86–1.59%), Planctomycetes (0.67–1.07%) (Figure 7A, Supplementary Table S1). The predominant genera with an average relative abundance (> 2%) were *Bradyrhizobium* (4.75–9.21%), *Enterobacter* (0.65–10.43%), *Sphingomonas* (2.91–6.31%), *Pseudomonas* (1.90–6.36%), *Ralstonia* (2.52–6.78%), *Massilia* (2.33–5.13%), *Streptomyces* (2.13–4.07%), *Rhizobium* (2.39–2.95%), *Microbacterium* (0.44–5.60%), *Mesorhizobium* (1.67–3.43%), *Sphingopyxis* (0.90–3.51%) (Figure 7B, Supplementary Table S2). Among the top 30 genera, the relative abundance of 11 and 2 genera were significantly increased and decreased, respectively, after the application of MgO NPs (compared with CK) (Supplementary Table S2). Compared with F1000, the relative abundance of 9 and 12 genera was significantly increased and decreased in MgO NPs treatments, respectively (Supplementary Table S2). The application of MgO NPs (500, 1,500, and 2,500 mg/L) significantly increased the relative abundance of *Pseudomonas* (85.25, 45.29, 10.50%, and 220.87, 151.65, 91.39%), *Sphingopyxis* (83.44, 8.59, 106.91% and 193.13, 73.52, 230.64%), *Acidovorax* (−28.04, 9.83, 19.18% and 67.99, 156.39, 178.24%), Var*iovorax* (17.10, 22.31, 62.60% and − 15.58, −11.82, 17.22%), and *Bosea* (33.59, 13.11, 3.74% and 91.59, 62.22, 48.78%) compared with CK and F1000, respectively. Eleven genera (*Bradyrhizobium*, *Sphingomonas*, *Ralstonia*, *Massilia*, *Mesorhizobium*, *Burkholderia*, *Caulobacter*, *Paraburkholderia*, *Mycobacterium*, *Azospirillum*, *Pseudolabrys*) showed the highest relative abundance in F1000 treatment.

### 3.7 Effect of MgO NPs on microbial functions

**Figure 8:**
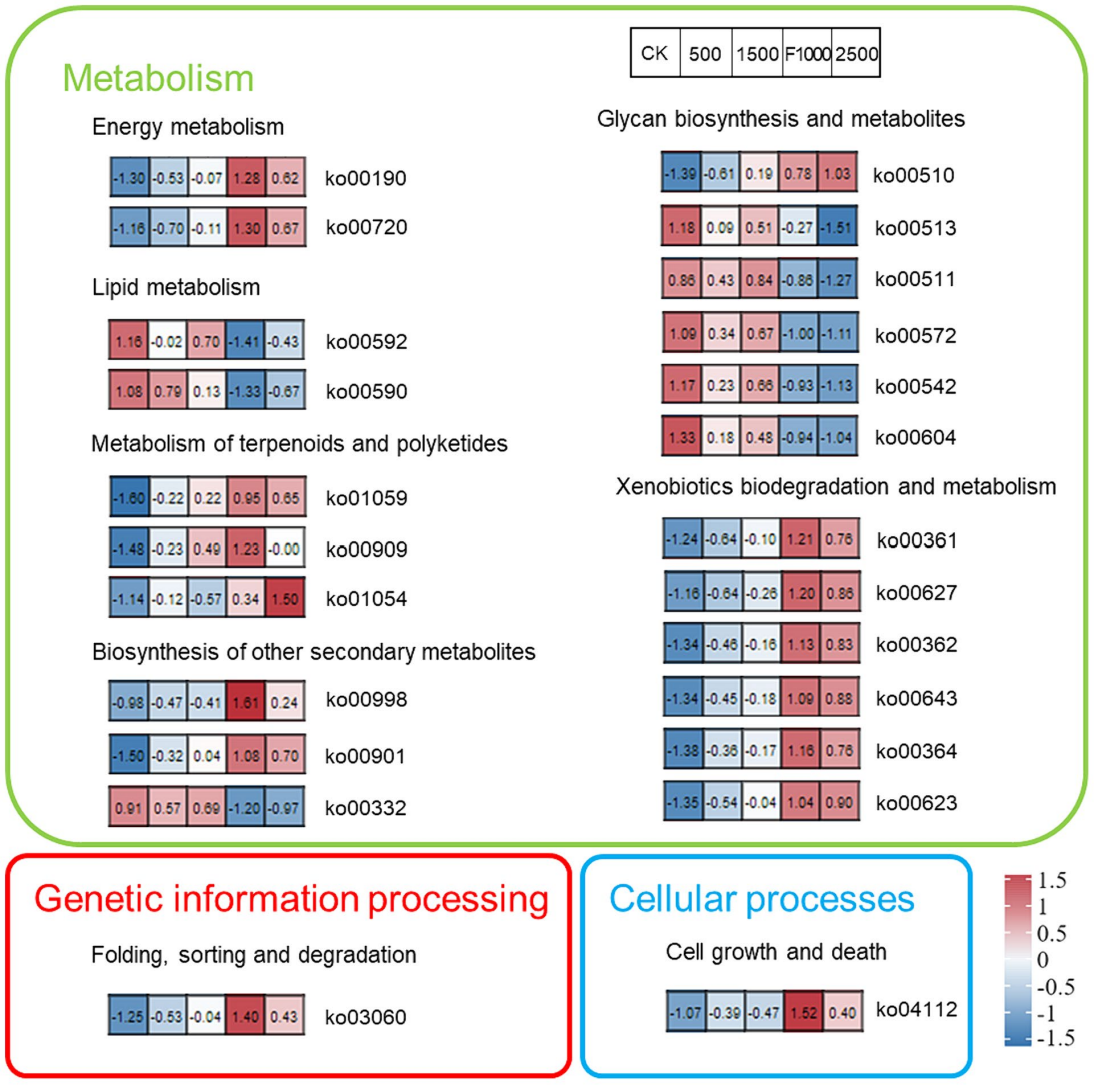
The heatmap showed the KEGG pathways significantly enriched by differentially abundant genes in all treatments. Each section represents the value of this function in each treatment, and the value was transformed into a Z-score. CK, F1000, 500, 1,500, and 2,500 represent water treatment, 1,000-fold dilution of fluazinam treatment, 500, 1,500, and 2,500 mg/L concentration of MgO NPs treatment after the inoculation of *P. brassicae*, respectively.

The KEGG function analysis helped us to further explore the impact of MgO NPs on rhizosphere microbiota functions (Figure 8). After comparing the significant differences of rhizosphere microbiota functions in different treatments, the top 24 significantly changed functions were screened for mapping. The results showed that the rhizosphere microbiota functions in high concentration of MgO NPs (2,500 mg/L) treatment was consistent with that of in F1000 treatment. The rhizosphere microbiota functions in low concentration of MgO NPs (500 and 1,500 mg/L) treatments was more consistent with that of in CK. Overall, plenty of functions associated with metabolism significantly changed in different treatments. Fourteen functions with metabolism enriched sharply in F1000 and MgO NPs (2,500 mg/L) treatment. Twelve functions with metabolism enriched in MgO NPs (1,500 mg/L) compared with CK. Eleven functions enriched gradually with the increase of concentration of MgO NPs treatments. Meanwhile, protein export (ko03060, the function associated with genetic information processing), and cell cycle-Caulobacter (ko04112, the function associated with cellular processes), had enriched in different degrees in MgO NPs and F1000 treatments compared with CK. On the contrary, eight functions associated with metabolism had decreased in MgO NPs and F1000 treatments compared with CK. Therefore, we speculated that a high concentration of MgO NPs might represent more influence on rhizosphere microbiota functions, and all concentrations of MgO NPs disturbed mainly metabolism.

## 4 Discussion

### 4.1 Effect of MgO NPs on clubroot control and plant growth

Magnesium oxide nanoparticles, as an environmentally friendly and non-toxic material, have been proven to have good greenhouse control effect on several plant diseases (Imada et al., 2016; Cai et al., 2018; Chen et al., 2020). Cai et al. (2018) found that the control efficacy of 250 mg/L MgO NPs (50 mL) on tobacco bacterial wilt was 36.5%. Chen et al. (2020) found that the control efficacy of 500 mg/L MgO NPs (20 mL) on tobacco black shank and black root rot were 50.2 and 62.1%, respectively. In their experiment period, the plants were irrigated with MgO NPs suspension only once. In this study (Figure 2), the efficacy of 500 mg/L MgO NPs (10 mL) on clubroot was 54.92%, and the plants were irrigated with MgO NPs suspension twice. When the concentration of MgO NPs was increased to 1,500 and 2,500 mg/L, there was no significant improvement in the control efficacy of clubroot. However, in the pre-experiment, we used 1,555 mg/L MgO NPs suspension to irrigate the plants three times, achieving a control efficacy of 76.4% (data not shown). Therefore, we believe that not only the concentration of nanomaterials, but also the application time and the quantity of nanomaterials may affect their effectiveness in preventing plant diseases. For *P. brassicae*, there may be a short cycle during the infection process (Buczacki and Clay, 1984; Liu et al., 2020) to rapidly increase population and accelerate the infection process. So, multiple application of nanomaterials may be more effective. The control effect of lower concentration and higher frequency of MgO NPs on clubroot remains to be explored, which can provide a basis for efficient utilization of MgO NPs. This study proves for the first time that nanomaterials also have the potential to control cruciferous clubroot.

Numerous studies have shown that nanomaterials can significantly promote plant growth (Arora et al., 2012; Nair, 2016), and its mechanism of action is multifaceted, including enhancing photosynthesis (Liu et al., 2023; Mehmood et al., 2023), improving the leaf or rhizosphere microbial communities (Karnwal et al., 2023; Liu et al., 2023), alleviating oxidative stress and enhancing antioxidant enzyme activity (Wu et al., 2023), and increasing the availability of nutrients in plants (Ahmed et al., 2022), regulating plant hormones and metabolism (Kang et al., 2023), and so on. The results of this study was consistent with part of previous studies. This study suggested that MgO NPs treatment could also promote the growth of tumorous stem mustard to a certain extent, because, there were no significant difference in plant growth between MgO NPs treatments (medium severity of clubroot) and F1000 treatment (lower severity of clubroot) (Figure 2, Supplementary Figure S1). We found that available phosphorus and potassium content in soil were significantly increased after the application of MgO NPs (compared with the F1000 treatment) (Figure 4). The Mg supply was also improved (Figure 4). In addition, soil enzyme activity and antioxidant enzyme activity in plant root tissues were significantly stimulated (Figure 5). The relative abundance of beneficial microorganisms in the rhizosphere also significantly increased, and genes related to plant growth promoting (PGP) traits and KEGG pathways were significantly enriched (Figures 7, 8). In summary, MgO NPs promoted the growth of tumorous stem mustard by improving soil nutrient conditions, reducing oxidative stress, and increasing the relative abundance of beneficial microorganisms. Finally, the enhancement of plant growth is also beneficial to enhancing its resistance to diseases. However, how MgO NPs are absorbed and utilized by plants, their accumulation in the root tissue, and their transportation in plants, as well as whether these processes affect the physiological, biochemical, and metabolic processes, and the expression of related genes, all await further exploration. These will help to unveil the deeper mechanisms of MgO NPs on promoting plant growth and disease resistance, and provide data for evaluating their food safety.

### 4.2 Mgo NPs may enhance the resistance of plant to clubroot by stimulating the growth of beneficial microorganisms, regulating rhizosphere microbial communities, and improving soil metabolic functions

The positive impact of healthy soil microbial communities on crop growth, soil fertility, and growth-regulating substances, as well as their ability to suppress soil-borne pathogens, are essential for sustainable agriculture (Saraiva et al., 2020; Karnwal et al., 2023; Williams et al., 2023). The stability of soil microorganisms is vulnerable to external environments, and the impact of nanomaterials can be positive or negative, depending on their type, concentration, and so on (Jośko et al., 2019; Yan et al., 2020; Samarajeewa et al., 2021; Liu et al., 2023). In this study, MgO NPs significantly improved the α-diversity of the rhizosphere microbiota of tumorous stem mustard, reversing the decrease in α-diversity caused by the invasion of *P. brassicae* (Figure 6). However, F1000 treatment, while effective in controlling clubroot, posed a serious threat to soil microbial α-diversity, posing a challenge to soil health and sustainable agricultural development. Similar to our results, Liu et al. (2023) found that nano-Fe_3_O_4_ can significantly increase bacterial diversity and abundance in the phylloplane under the contamination of microplastic pollution. Yan et al. (2020) found that archaeal richness and diversity under Ag NPs exposure increased. However, some studies found that nanomaterials may not affect or reduce the α-diversity. Yan et al. (2020) found that Ag NPs reduce the soil bacterial diversity. Moll et al. (2017) found that TiO_2_ NPs do not affect the α-diversity of soil prokaryotes and fungi. However, MgO NPs could improve soil microbial diversity, which may also be related to the importance of magnesium in soil microorganisms.

At the phylum level, the relative abundances of the 11 bacterial phyla and two fungal phyla treated with MgO NPs at varying concentrations were comparable to those of the CK. Nevertheless, Proteobacteria remains the most abundant phylum in all treatments, consistent with previous research findings (Lebreton et al., 2019; Wang et al., 2020; Liao et al., 2022; Xi et al., 2023). However, at the genus level, significant disparities emerged between the MgO NPs treatments and the CK. Notably, the top 30 genera exclusively consisted of bacteria. Researches have established that bacteria are more vulnerable to the infection of *P. brassicae* than fungi in rhizosphere soil, and *P. brassicae* tends to engage in direct or indirect interactions with bacteria rather than fungi (Lebreton et al., 2019; Xi et al., 2023). Therefore, our primary focus was on examining the alterations in bacterial genera brought about by exposure to MgO NPs.

The effect of MgO NPs on genera composition could also partially explain its role in promoting plant growth and resistance to clubroot. In this study, MgO NPs treatments significantly increased the relative abundance of *Pseudomonas*, *Sphingopyxis*, *Acidovorax*, Var*iovorax*, *Bosea* in soil (Supplementary Table S2). Among these genera, *Pseudomonas*, as a biological control agent for pests and diseases, has been widely utilized in enhancing and facilitating crop production (Navarro-Monserrat and Taylor, 2023). Notably, it exhibited the highest relative abundance in the 500 and 1,500 mg/L MgO NPs treatments (Supplementary Table S2). *P. putida*, which is one of the most studied species beneficial for plant development and/or biological control of plant pathogens (Navarro-Monserrat and Taylor, 2023), was the biomarker specie in the 500 mg/L MgO NPs treatment (Supplementary Figure S2). *Pseudomonas* has also been proved play an important role in clubroot disease suppression (Zhang et al., 2022). *Sphingopyxis*, which was stimulated by the carbon nanosol (CNS) nanomaterial, had been demonstrated to enhance plant growth (Cheng et al., 2023). It exhibited the highest relative abundance in the 500 and 1,500 mg/L MgO NPs treatments (Supplementary Table S2). *Acidovorax*, which was identified as a biomarker in the 2,500 mg/L MgO NPs treatment (Supplementary Figure S2), has been reported to produce secondary metabolites and hormones that promote plant growth and compete with pathogens, in some strains (Siani et al., 2021). Some strains in *Variovorax* and *Bosea* also exhibited the ability to promote plant growth and combat plant pathogens (Bruisson et al., 2019; Khoury et al., 2021; Ma et al., 2023). Other genera of microorganisms, such as *Candidatus*_*Koribacter*, *Nitrospira* (Kang et al., 2023), which were reported by Saraiva et al. (2020) to be the dominant in soils with less severe clubroot disease, were significantly enriched after the application of MgO NPs (2,500 mg/L) compared with CK (Supplementary Table S2).

The shift in microbial community composition inevitably alters soil microbial function. Our results (Supplementary Tables S3–S5) revealed that the application of MgO NPs significantly increased the enrichment of genes related to plant growth promoting (PGP) traits, such as those involved in the indole-3-acetic acid (IAA) biosynthesis, the nitrogen cycle, and the phosphorus solubilization. Conversely, genes related to siderophore production were significantly reduced. This phenomena was similar to the results of Cheng et al. (2023). The notably enrichment of these genes may be linked to the improvement of soil nutrients and the promotion of plant growth upon the application of MgO NPs. In addition, we observed that the enrichment of CAT enzyme genes in MgO NPs and F1000 treatments, was significantly lower than that in CK, aligning with the results of CAT enzyme activity in soil. When comparing the KEGG enrichment pathways, it was found that among the 14 metabolic functions significantly enriched in the MgO NPs and F1000 treatments, oxidative phosphorylation (Usmani et al., 2019) and prokaryotic carbon fixation (Overholt et al., 2022) provided energy and nutrients for the activities of soil microorganisms. Indole alkaloids can function as signaling molecules and play a role in the interactions between plants and the environment (Khan et al., 2019). Furthermore, the enrichment of biosynthesis of various antibiotics function may also be one of the reasons why MgO NPs aided tumorous stem mustard in combating clubroot (He et al., 2023). In summary, MgO NPs enhanced plant growth and aided in the resistance to clubroot by recruiting beneficial microorganisms and regulating soil microbial metabolic functions (Qi et al., 2022).

### 4.3 Mgo NPs stimulated the activity of plant antioxidant enzymes and promoted SA accumulation to resist the infection of *P. brassicae*

SA and antioxidant enzymes play an important role in resisting plant disease. SA, as a signal molecule for plant defense response, induces plant resistance through various pathways. Antioxidant enzymes, which can eliminate oxidative stress caused by pathogens, can enhance plant resistance to pathogens. Shetehy et al. (2021) found that low-concentration of silica nanoparticles induce systemic acquired resistance (SAR) in *Arabidopsis thaliana* through an SA-dependent defense pathway to resist infection by *Pseudomonas syringae*, without directly affecting the growth of the pathogen. Ahmed et al. (2022) demonstrated that chitosan-iron nanocomposite controlled bacterial leaf blight by regulating the defense response and nutritional status of rice plants. Kang et al. (2023) proved that nano-selenium enhances melon resistance to *Podosphaera xanthii* by enhancing the antioxidant capacity and promoting hormone signaling pathways. This study found that MgO NPs treatment significantly increased SA content in the root of tumorous stem mustard, and also significantly increased the antioxidant enzyme activity (Figure 3), which was similar to the results of those studies. It is speculated that MgO NPs may also induce SAR in tumorous stem mustard through an SA-dependent defense pathway to resist the infection of *P. brassicae*. However, further verification is needed to clarify the anti-pathogenic mechanism of the nanomaterials, laying the foundation for the efficient utilization of nanomaterials in the prevention of plant diseases.

### 4.4 The direct effect of MgO NPs on *P. brassicae* required further exploration

Nanomaterials, with their superior physical and chemical properties, can inhibit the activity of pathogenic microorganisms by directly contacting their cells, causing cell damage and triggering the accumulation of reactive oxygen species. The results of Chen et al. (2020) showed that MgO NPs directly in contact with tobacco black shank and black root rot fungi caused changes in fungal cell morphology, zeta potential disorder, and accumulation of reactive oxygen species resulting in oxidative stress, which inhibited the growth of pathogenic fungi. Abdel-Aziz et al. (2020) also demonstrated that MgO NPs can significantly inhibit the growth of *Fusarium oxysporum* hyphae and biofilm formation, causing severe morphological changes, significantly disrupting the integrity of fungal biofilms, thereby achieving antifungal effects. However, this study cannot rule out the direct effect of MgO NPs on *P. brassicae*, and further exploration is needed to investigate its impact on the germination of *P. brassicae* resting spores and whether it causes physical damage to resting spores, in order to clarify the mechanism of action on *P. brassicae* and fill the gaps in this research field.

## 5 Conclusion

We found that MgO NPs could effectively reduce clubroot severity on tumorous stem mustard. It also promoted plant growth to a certain extent, significantly altered soil nutrient status, soil enzyme activity, and plant defense enzyme activity, as well as the structure and functions of rhizosphere microbial community. Specifically, the promoting effect of MgO NPs on plant growth might be related to the improvement of available phosphorus, potassium, and magnesium content in the soil, as well as the corresponding increase in enzyme activities such as alkaline phosphatase and sucrase. Furthermore, it was accompanied by a significant enrichment of beneficial microorganisms in the rhizosphere, such as *Pseudomonas*, *Sphingopyxis* and so on, as well as an enrichment of genes related to plant growth promoting traits and KEGG pathways. The improvement of plant growth, the increase of plant defense enzyme activity, the enrichment of beneficial microorganisms in the rhizosphere, and the enrichment of the biosynthesis of various antibiotics function might all contribute to the prevention of clubroot by MgO NPs. Therefore, the beneficial effect of MgO NPs on clubroot of tumorous stem mustard appears to be achieved through their impact on multiple pathways.

## Data availability statement

The datasets presented in this study can be found in online repositories. The names of the repository/repositories and accession number(s) can be found in https://www.ncbi.nlm.nih.gov/bioproject/PRJNA1056493.

## Ethics statement

The study was approved by Yangtze Normal University. Participants provided signed informed consent. All methods were carried out in accordance with relevant guidelines and regulations.

## Author contributions

JL: Conceptualization, Funding acquisition, Investigation, Writing – original draft, Writing – review & editing. ZY: Investigation, Writing – original draft, Writing – review & editing. XW: Investigation, Writing – original draft. TC: Investigation, Writing – review & editing. KQ: Conceptualization, Resources, Writing – review & editing. YC: Investigation, Writing – review & editing. AR: Investigation, Writing – review & editing. CZ: Investigation, Writing – review & editing. YL: Investigation, Writing – review & editing. DW: Funding acquisition, Project administration, Supervision, Writing – review & editing. LP: Funding acquisition, Project administration, Supervision, Writing – review & editing.

## Footnotes

^1^ http://www.omicshare.com/tools
