# Supplementary figures and images for: Magnesium oxide nanoparticles reduce clubroot by regulating plant defense response and rhizosphere microbial community of tumorous stem mustard (Brassica juncea var. tumida)
Source: Front Microbiol. 2024 Mar 20;15:1370427. doi: 10.3389/fmicb.2024.1370427 (PMC10989686; doi:10.3389/fmicb.2024.1370427)

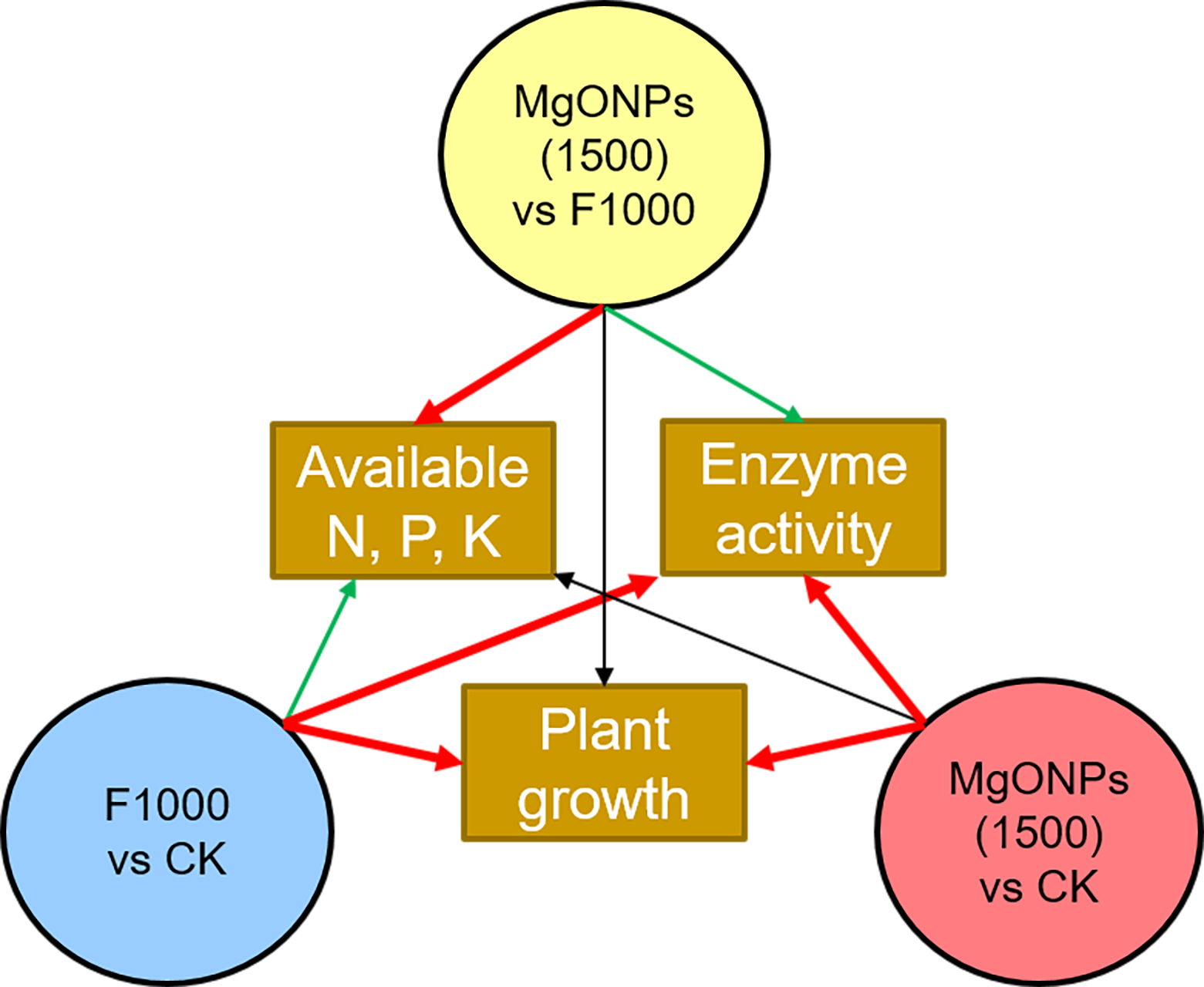

Supplement: Supplementary file 1 [file Image_1.TIF]

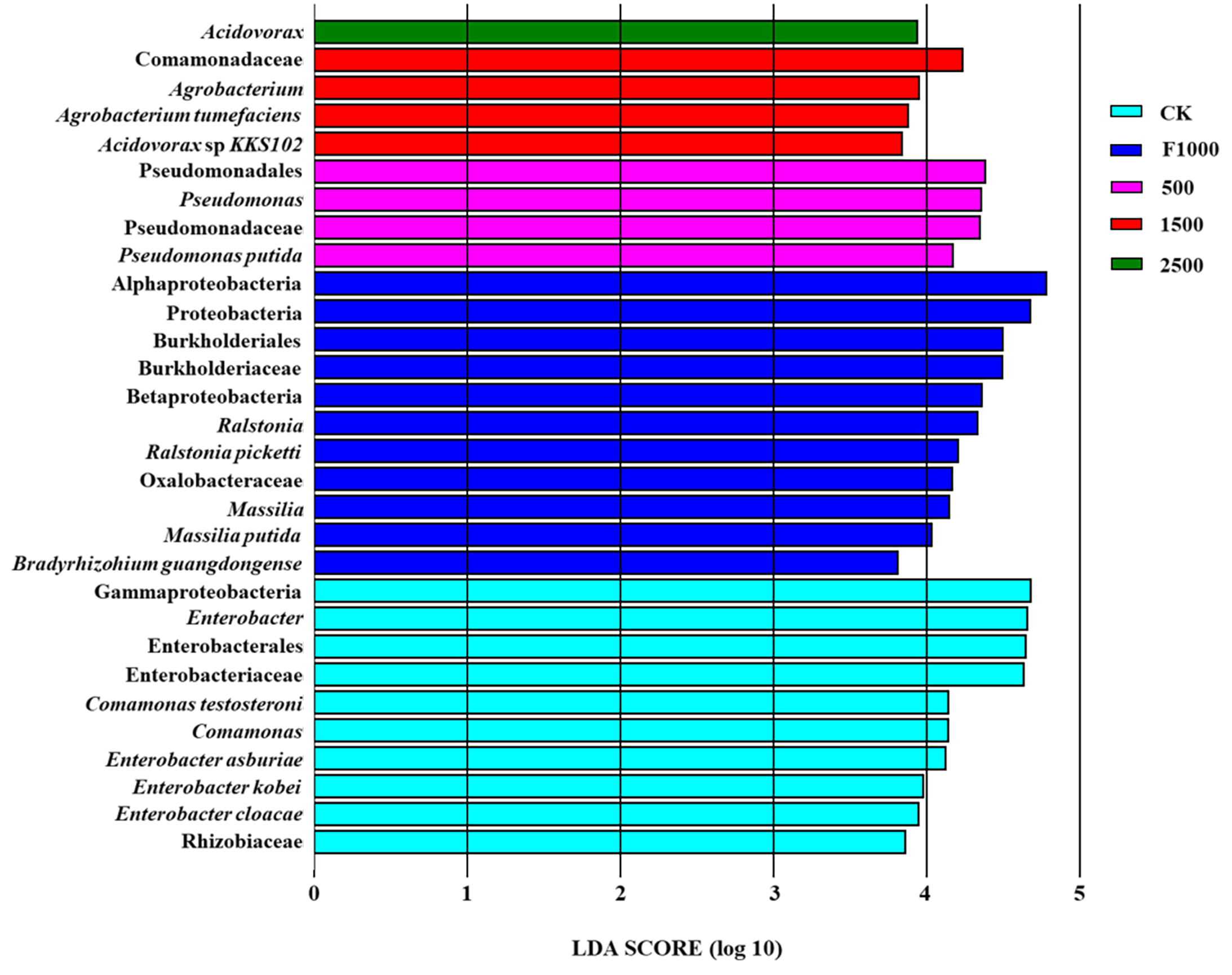

Supplement: Supplementary file 2 [file Image_2.TIF]
