# Supplementary material for: Magnesium oxide nanoparticles reduce clubroot by regulating plant defense response and rhizosphere microbial community of tumorous stem mustard (Brassica juncea var. tumida)
Source: Front Microbiol. 2024 Mar 20;15:1370427. doi: 10.3389/fmicb.2024.1370427 (PMC10989686; doi:10.3389/fmicb.2024.1370427)
Supplement: Supplementary file 3 [file Table_1.DOCX]

**Table S1 Relative abundances of bacteria at the phylum level.**

| **Phylum** | **Relative abundance (%)** | | | | |
| --- | --- | --- | --- | --- | --- |
|  | **CK** | **F1000** | **500** | **1500** | **2500** |
| Proteobacteria | 68.67 ± 0.38c | 75.35 ± 0.34a | 70.36 ± 0.65c | 69.40 ± 1.03c | 73.20 ± 0.19b |
| Actinobacteria | 17.43 ± 0.30a | 16.35 ± 0.39b | 16.57 ± 0.18ab | 17.05 ± 0.51ab | 16.58 ± 0.14ab |
| Bacteroidetes | 9.40 ± 0.98a | 1.81 ± 0.03b | 7.54 ± 0.55a | 8.17 ± 0.64a | 3.95 ± 0.19a |
| Firmicutes | 1.27 ± 0.08a | 1.60 ± 0.09a | 1.50 ± 0.11a | 1.67 ± 0.18a | 1.47 ± 0.15a |
| Acidobacteria | 0.86 ± 0.08b | 1.59 ± 0.01a | 1.11 ± 0.01b | 1.04 ± 0.03b | 1.25 ± 0.08ab |
| Planctomycetes | 0.67 ± 0.08ab | 1.04 ± 0.01a | 0.83 ± 0.01b | 0.81 ± 0.02b | 1.07 ± 0.08ab |
| Ascomycota | 0.55 ± 0.05ab | 0.49 ± 0.03b | 0.69 ± 0.06a | 0.45 ± 0.05b | 0.55 ± 0.03ab |
| Gemmatimonadetes | 0.17 ± 0.02b | 0.32 ± 0.01a | 0.24 ± 0.00b | 0.24 ± 0.01b | 0.47 ± 0.03a |
| Verrucomicrobia | 0.15 ± 0.01e | 0.22 ± 0.00b | 0.18 ± 0.00d | 0.20 ± 0.01c | 0.30 ± 0.01a |
| Euryarchaeota | 0.17 ± 0.02c | 0.25 ± 0.01a | 0.20 ± 0.01bc | 0.19 ± 0.01bc | 0.22 ± 0.01ab |
| Cyanobacteria | 0.15 ± 0.01ab | 0.23 ± 0.00a | 0.18 ± 0.01ab | 0.18 ± 0.00b | 0.21 ± 0.01ab |
| Deinococcus-Thermus | 0.12 ± 0.01ab | 0.20 ± 0.00a | 0.14 ± 0.00b | 0.15 ± 0.00b | 0.18 ± 0.01ab |
| Chloroflexi | 0.11 ± 0.02ab | 0.18 ± 0.00a | 0.14 ± 0.00b | 0.14 ± 0.01ab | 0.17 ± 0.01ab |
