# Supplementary material for: Magnesium oxide nanoparticles reduce clubroot by regulating plant defense response and rhizosphere microbial community of tumorous stem mustard (Brassica juncea var. tumida)
Source: Front Microbiol. 2024 Mar 20;15:1370427. doi: 10.3389/fmicb.2024.1370427 (PMC10989686; doi:10.3389/fmicb.2024.1370427)
Supplement: Supplementary file 4 [file Table_2.DOCX]

**Table S2 Relative abundance of microorganisms at the genus level**

| **Genus** | **Relative abundance (%)** | | | | |
| --- | --- | --- | --- | --- | --- |
|  | **CK** | **F1000** | **500** | **1500** | **2500** |
| *Bradyrhizobium* | 5.51 ± 0.61b | 8.65 ± 0.28a | 5.70 ± 0.09b | 6.14 ± 0.33b | 6.67 ± 0.40b |
| *Enterobacter* | 9.13 ± 0.99a | 0.78 ± 0.08b | 4.78 ± 0.09a | 4.18 ± 0.10a | 3.80 ± 0.35a |
| *Sphingomonas* | 3.06 ± 0.09d | 6.05 ± 0.13a | 3.90 ± 0.06c | 3.18 ± 0.15d | 4.49 ± 0.10b |
| *Pseudomonas* | 3.30 ± 0.17b | 1.91 ± 0.01c | 6.12 ± 0.15a | 4.80 ± 0.08a | 3.65 ± 0.40abc |
| *Ralstonia* | 2.69 ± 0.09c | 6.48 ± 0.22a | 2.81 ± 0.08c | 3.65 ± 0.04b | 2.86 ± 0.09c |
| *Massilia* | 2.68 ± 0.04de | 4.98 ± 0.11 a | 2.88 ± 0.04cd | 3.67 ± 0.10b | 2.50 ± 0.09e |
| *Streptomyces* | 2.44 ± 0.26abc | 3.97 ± 0.05a | 3.02 ± 0.02c | 3.01 ± 0.05bc | 3.54 ± 0.06ab |
| *Rhizobium* | 2.90 ± 0.02a | 2.69 ± 0.13abc | 2.76 ± 0.11ab | 2.55 ± 0.07bc | 2.46 ± 0.04c |
| *Microbacterium* | 5.09 ± 0.42ab | 0.45 ± 0.01c | 2.57 ± 0.04b | 3.21 ± 0.40bc | 1.48 ± 0.23bc |
| *Mesorhizobium* | 1.99 ± 0.13b | 3.02 ± 0.21a | 1.76 ± 0.01b | 1.89 ± 0.13b | 2.14 ± 0.06b |
| *Sphingopyxis* | 1.57 ± 0.05b | 0.98 ± 0.06c | 2.88 ± 0.02a | 1.70 ± 0.05b | 3.25 ± 0.18a |
| *Burkholderia* | 2.00 ± 0.14ab | 2.36 ± 0.01a | 1.80 ± 0.04b | 1.74 ± 0.06b | 1.64 ± 0.01b |
| *Flavobacterium* | 2.94 ± 0.37ab | 0.40 ± 0.02b | 1.56 ± 0.13a | 3.03 ± 0.32a | 0.67 ± 0.04ab |
| *Acidovorax* | 1.82 ± 0.06c | 0.78 ± 0.02e | 1.31 ± 0.02d | 2.00 ± 0.06b | 2.17 ± 0.04a |
| *Mucilaginibacter* | 2.62 ± 0.25ab | 0.36 ± 0.01c | 2.12 ± 0.09a | 1.52 ± 0.13ab | 1.05 ± 0.09bc |
| *Caulobacter* | 1.16 ± 0.02c | 1.78 ± 0.03a | 1.39 ± 0.02bc | 1.14 ± 0.07bc | 1.49 ± 0.01b |
| *Comamonas* | 2.39 ± 0.06a | 0.33 ± 0.00d | 1.64 ± 0.02b | 1.57 ± 0.04b | 0.72 ± 0.03c |
| *Variovorax* | 0.99 ± 0.02d | 1.37 ± 0.04b | 1.16 ± 0.03c | 1.2 ± 0.06c | 1.61 ± 0.06a |
| *Bosea* | 1.13 ± 0.02c | 0.79 ± 0.01d | 1.51 ± 0.01a | 1.28 ± 0.01b | 1.18 ± 0.01c |
| *Sphingobium* | 1.20 ± 0.04ab | 1.19 ± 0.02ab | 1.25 ± 0.01a | 0.94 ± 0.02c | 1.14 ± 0.01b |
| *Sphingobacterium* | 1.67 ± 0.20a | 0.09 ± 0.01b | 1.96 ± 0.21a | 1.43 ± 0.14a | 0.47 ± 0.02a |
| *Agrobacterium* | 1.30 ± 0.07ab | 0.30 ± 0.01b | 1.16 ± 0.01c | 1.59 ± 0.04a | 1.28 ± 0.06ab |
| *Micromonospora* | 0.63 ± 0.07d | 1.14 ± 0.07b | 0.93 ± 0.03c | 1.07 ± 0.01bc | 1.72 ± 0.10a |
| *Delftia* | 1.04 ± 0.04a | 0.16 ± 0.02b | 0.92 ± 0.03a | 1.70 ± 0.23ab | 0.38 ± 0.02b |
| *Paraburkholderia* | 0.69 ± 0.02b | 1.09 ± 0.01a | 0.67 ± 0.01b | 0.72 ± 0.04b | 0.69 ± 0.02b |
| *Mycobacterium* | 0.59 ± 0.04c | 0.87 ± 0.04a | 0.71 ± 0.01b | 0.68 ± 0.02bc | 0.72 ± 0.03b |
| *Azospirillum* | 0.58 ± 0.00b | 0.84 ± 0.01a | 0.58 ± 0.01b | 0.61 ± 0.03b | 0.56 ± 0.01b |
| *Pseudolabrys* | 0.44 ± 0.04d | 0.91 ± 0.02a | 0.54 ± 0.02c | 0.56 ± 0.02c | 0.72 ± 0.04b |
| *Novosphingobium* | 0.51 ± 0.01b | 0.59 ± 0.01b | 0.57 ± 0.01b | 0.53 ± 0.01b | 0.94 ± 0.03a |
| *Nocardioides* | 0.53 ± 0.02c | 0.67 ± 0.03a | 0.66 ± 0.01ab | 0.59 ± 0.02bc | 0.64 ± 0.03ab |
| *Candidatus_Koribacter* | 0.12 ± 0.012c | 0.24 ± 0.001a | 0.16 ± 0.002c | 0.15 ± 0.004c | 0.19 ± 0.015b |
| *Nitrospira* | 0.05 ± 0.008b | 0.08 ± 0.005a | 0.06 ± 0.002b | 0.06 ± 0.003b | 0.08 ± 0.008a |
